# Muscleblind-like 1 is required for normal heart valve development *in vivo*

**DOI:** 10.1186/s12861-015-0087-4

**Published:** 2015-10-15

**Authors:** Ryan J. Coram, Samantha J. Stillwagon, Anuradha Guggilam, Michael W. Jenkins, Maurice S. Swanson, Andrea N. Ladd

**Affiliations:** Department of Cellular & Molecular Medicine, Lerner Research Institute, 9500 Euclid Ave. NC10, Cleveland Clinic, Cleveland, OH 44195 USA; Department of Pediatrics, Case Western Reserve University School of Medicine, Cleveland, OH 44106 USA; Department of Molecular Genetics & Microbiology, College of Medicine, Center for NeuroGenetics and the Genetics Institute, University of Florida, Gainesville, FL 32610 USA; Present Address: Ohio University Heritage College of Osteopathic Medicine, Athens, OH 45701 USA; Present Address: Department of Obstetrics and Gynecology, Women’s Health Institute, Cleveland Clinic, Cleveland, OH 44195 USA

**Keywords:** Muscleblind-like 1, Transforming growth factor β, Epithelial-mesenchymal transition, Endocardial cushions, Heart valves, Mouse

## Abstract

**Background:**

Development of the valves and septa of the heart depends on the formation and remodeling of the endocardial cushions in the atrioventricular canal and outflow tract. These cushions are populated by mesenchyme produced from the endocardium by epithelial-mesenchymal transition (EMT). The endocardial cushions are remodeled into the valves at post-EMT stages via differentiation of the mesenchyme and changes in the extracellular matrix (ECM). Transforming growth factor β (TGFβ) signaling has been implicated in both the induction of EMT in the endocardial cushions and the remodeling of the valves at post-EMT stages. We previously identified the RNA binding protein muscleblind-like 1 (MBNL1) as a negative regulator of TGFβ signaling and EMT in chicken endocardial cushions *ex vivo*.  Here, we investigate the role of MBNL1 in endocardial cushion development and valvulogenesis in *Mbnl1*^∆E3/∆E3^ mice, which are null for MBNL1 protein.

**Methods:**

Collagen gel invasion assays, histology, immunohistochemistry, real-time RT-PCR, optical coherence tomography, and echocardiography were used to evaluate EMT and TGFβ signaling in the endocardial cushions, and morphogenesis, ECM composition, and function of the heart valves.

**Results:**

As in chicken, the loss of MBNL1 promotes precocious TGFβ signaling and EMT in the endocardial cushions. Surprisingly, this does not lead to the production of excess mesenchyme, but later valve morphogenesis is aberrant. Adult *Mbnl1*^*∆E3/∆E3*^ mice exhibit valve dysmorphia with elevated TGFβ signaling, changes in ECM composition, and increased pigmentation. This is accompanied by a high incidence of regurgitation across both inflow and outflow valves. *Mbnl1*^*∆E3/∆E3*^ mice also have a high incidence of ostium secundum septal defects accompanied by atrial communication, but do not develop overt cardiomyopathy.

**Conclusions:**

Together, these data indicate that MBNL1 plays a conserved role in negatively regulating TGFβ signaling, and is required for normal valve morphogenesis and homeostasis *in vivo*.

**Electronic supplementary material:**

The online version of this article (doi:10.1186/s12861-015-0087-4) contains supplementary material, which is available to authorized users.

## Background

Valve defects are among the most common congenital heart defects, and are associated with significant health problems and mortality throughout life [1]. Their exact incidence is unknown because many valve defects are not diagnosed until adulthood [2]. Aortic valve defects alone occur in 1-2 % of the population [3]. The prevalence of adults with valvular heart disease is increasing, due to improved surgical treatments and increases in lifespan that may be accompanied by valve degeneration [4]. Congenital valve defects and adult valve disease may not be distinct, since structural or biomechanical abnormalities in the valves may be present but not readily appreciated at birth. The heart valves form by remodeling embryonic structures called endocardial cushions. Outgrowth of the cushions occurs via localized expansion of extracellular matrix (ECM) between the endocardium and myocardium in the atrioventricular canal (AVC) and outflow tract (OFT), after which the cushions are cellularized by invasion of a subpopulation of endocardial cells undergoing epithelial-mesenchymal transition (EMT) [5]. At post-EMT stages, this cushion mesenchyme differentiates into valve interstitial cells, specialized fibroblasts that produce the highly stratified matrix of the mature valves [2, 3]. The mitral and tricuspid valves form from the AVC endocardial cushions, while the aortic and pulmonary valves arise from the OFT cushions [6].

Transforming growth factor β (TGFβ) signaling induces endocardial cushion EMT in both birds and mammals, although the roles of specific TGFβ paralogs differ [7–9]. In chick, TGFβ2 is required for activation of the endocardial cells, in which cell polarity and cell-cell contacts are lost, and TGFβ3 is required for invasion of the transformed mesenchymal cells into the cushion matrix [8, 9]. TGFβ1 is not expressed in the developing cushions. Both TGFβ2 and TGFβ3 are produced in the myocardium prior to the onset of EMT, and TGFβ3 is found in the endocardium during stages of active EMT [8, 10, 11]. Autocrine production of TGFβ3 in the cushion endocardium is essential for induction of invasive mesenchyme [11, 12]. In mouse, TGFβ3 is not expressed in the heart until E11.5, well after EMT is underway, and is restricted to the mesenchymal cells of the cushions [13]. No cardiac malformations have been found in *Tgfb3*-null mice [14, 15]. TGFβ2 has an expression pattern in the mouse heart that is similar to that of TGFβ3 in chick, and inhibition of TGFβ2 blocks invasion in mouse AVC explants [13, 16]. *Tgfb2*-null mice exhibit impaired EMT, hypocellular cushions, and a variety of valve and septal defects [7, 17, 18]. Thus, in mouse TGFβ2 plays a role orthologous to that of chick TGFβ3. TGFβ1 is also expressed in mouse AVC endocardium, and although it is not essential for endocardial cushion EMT *in vivo*, *Tgfb1*-null mice have disorganized valves [19].

We previously identified a role for muscleblind-like 1 (MBNL1) in the regulation of TGFβ-dependent EMT in the endocardial cushions in chick [20, 21]. MBNL1 is a member of the MBNL family of RNA binding proteins that regulate pre-mRNA alternative splicing, alternative polyadenylation, microRNA biogenesis, mRNA stability and localization [22–25]. We previously reported that in the embryonic chicken heart *MBNL1* transcripts are found in the atrial and ventricular myocardium, and in the endocardium of the AVC and OFT endocardial cushions during EMT and immediately prior to its onset [20, 21]. We demonstrated that knockdown of MBNL1 in chick AVC or OFT explants promotes invasive mesenchyme formation *ex vivo* [20, 21]. This effect is dependent upon TGFβ3, and is accompanied by precocious and elevated secretion of active TGFβ3 by the endocardial cells [20, 21]. Early exposure to excess TGFβ3 is sufficient to induce precocious and elevated invasion in AVC explants [20]. These data suggested that MBNL1 negatively regulates EMT in the endocardial cushions by limiting the levels and timing of inductive, autocrine TGFβ signaling.

In this study, we investigate the role of MBNL1 in mouse endocardial cushion development and valvulogenesis. We demonstrate that high expression of MBNL1 in the endocardial cushions is conserved in mouse. Using *Mbnl1*^*∆E3/∆E3*^ mice, which are null for MBNL1 protein, we show that loss of MBNL1 promotes invasive mesenchyme production in AVC explants *ex vivo* and precocious TGFβ signaling and EMT in the AVC *in vivo*, but does not lead to an excess in total mesenchyme production. In the fetal *Mbnl1*^*∆E3/∆E3*^ heart, however, valve morphogenesis is aberrant. In the adult heart, *Mbnl1*^*∆E3/∆E3*^ mice continue to exhibit valve dysmorphia, accompanied by elevated TGFβ signaling and changes in ECM composition. Significant regurgitation was observed across both inflow and outflow valves. *Mbnl1*^*∆E3/∆E3*^ mice also have a high incidence of ostium secundum atrial septal defects and increased valve pigmentation. Taken together, these data indicate that MBNL1 is required for normal heart valve development and function *in vivo*.

## Results

### MBNL1 is expressed in the endocardial cushions and mature heart valves

**Fig. 1:**
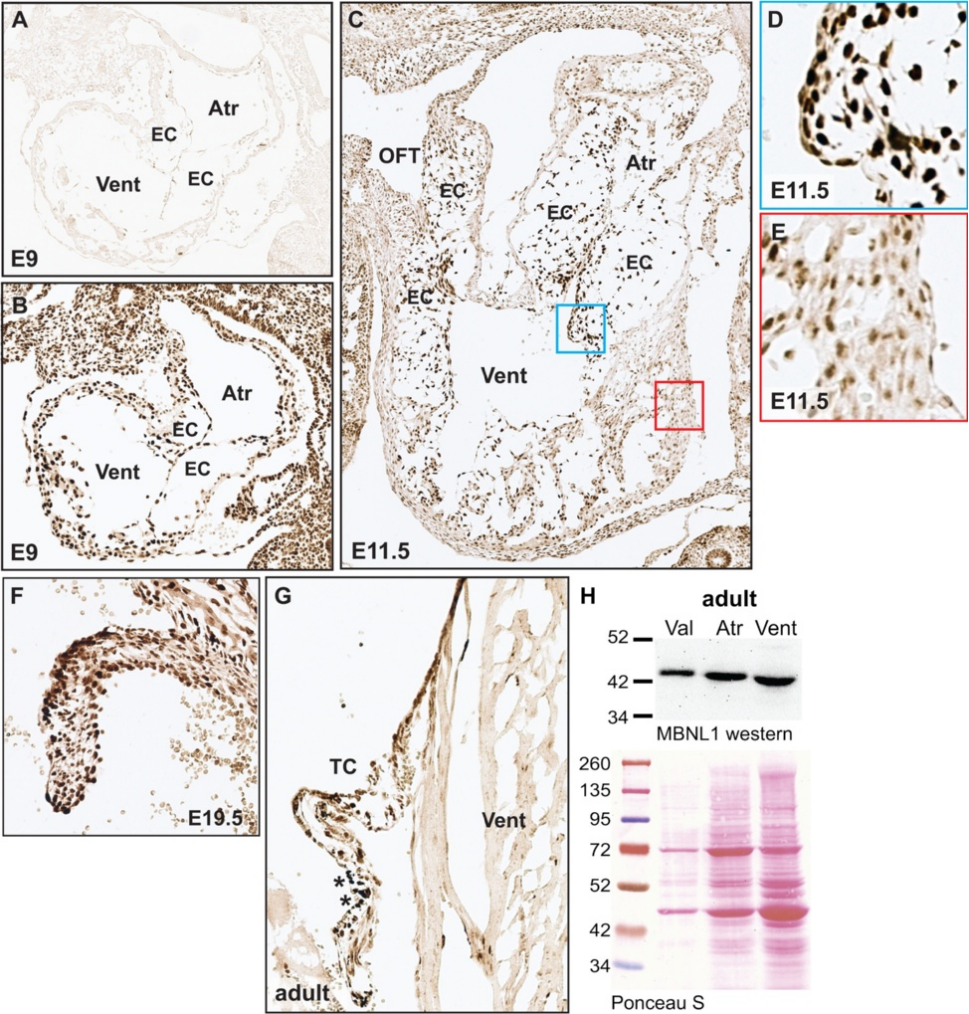
MBNL1 is highly expressed in the endocardial cushions and heart valves. Immunohistochemistry with an anti-MBNL1 antibody was performed on sagittal sections from wild type mouse embryos (**a**-**f**) and coronal sections of adult hearts (**g**). Representative sections from one of four embryos or one of two adult hearts are shown. **a** Close-up of the heart at E9 stained with the secondary antibody alone is shown as a negative control. **b** Close-up of the heart at E9 shows expression of MBNL1 in the early myocardium and endocardium of the nascent endocardial cushions just prior to the onset of EMT. **c** Close-up of the heart at E11.5 shows strong MBNL1 expression in the endocardial cushions of the AVC and OFT. Higher magnification views of the boxed regions in the endocardial cushion (**d**) and the ventricular wall (**e**) show intense nuclear staining in the cushion endocardial cells, but more diffuse staining in the myocardium. Intense nuclear MBNL1 staining persists in the fetal valves at E19.5 (**f**) and mature heart valves (**g**), while MBNL1 remains more diffusely distributed in the nucleus and cytoplasm in the myocardium. Asterisks highlight accumulations of melanin in the adult valve. **h** Western blot analysis confirms high MBNL1 expression in the adult heart, and suggests different cardiac tissues may express different MBNL1 isoforms. Protein integrity and loading were assessed by Ponceau S staining. A representative of three independent blots with similar results is shown. Atr = atrium, Vent = ventricle, EC = endocardial cushion, OFT = outflow tract, TC = tricuspid valve, Val = heart valves

To determine whether MBNL1 expression in the endocardial cushions is conserved in mammals, we performed immunohistochemistry on sections from mouse hearts at different stages of development. As in the chicken heart, MBNL1 was strongly detected in the myocardium and AVC endocardium at E9, prior to the onset of EMT (Fig. 1a, b). MBNL1 is broadly expressed throughout the embryo, but is not ubiquitous; for example, although MBNL1 protein is strongly detected in the ectodermal linings it is low or absent in the interior cephalic tissues of the mid-, fore-, and hindbrain (Additional file 1: Figure S1). At E11.5, when EMT is well underway, intense nuclear MBNL1 staining was observed in the endocardium and mesenchyme of the AVC and OFT cushions (Fig. 1c, d). Strong nuclear MBNL1 staining persists in the immature valves at late fetal stages (Fig. 1f) and in the mature valves of the adult heart (Fig. 1g). MBNL1 was also seen in the developing myocardium, where it is found in both the nucleus and cytoplasm (Fig. 1c, e, f). In the adult myocardium, MBNL1 staining is more diffuse and cytoplasmic (Fig. 1g), consistent with previous reports that MBNL1 is more strongly detected in cytoplasmic than nuclear extracts from whole hearts at postnatal stages [26, 27].

By western blot, MBNL1 is expressed in adult heart valves at levels comparable or higher than levels in the chambers (Fig. 1h). Interestingly, slightly different MBNL1 isoforms were detected in the different compartments of the heart, with the slowest migrating isoform in the valves and the fastest in the ventricles. Alternative splicing of *Mbnl1* transcripts can lead to the generation of several different MBNL1 protein isoforms, ranging in size from 35 to 43 kDa [28]. We and others previously reported that inclusion or skipping of exon 7 [28] affects the subcellular localization of MBNL1 protein such that the larger exon-included form is predominantly nuclear whereas the exon-skipped form is both nuclear and cytoplasmic [29–31]. Although preferential inclusion of exon 7 in the heart valves would explain both its strong nuclear localization and its slower migration, we found that exon 7 is in fact ~95 % skipped in adult mouse heart valves, atria, and ventricles (data not shown).

### Loss of MBNL1 has a transient stimulatory effect on EMT in the endocardial cushions *ex vivo*

**Fig. 2:**
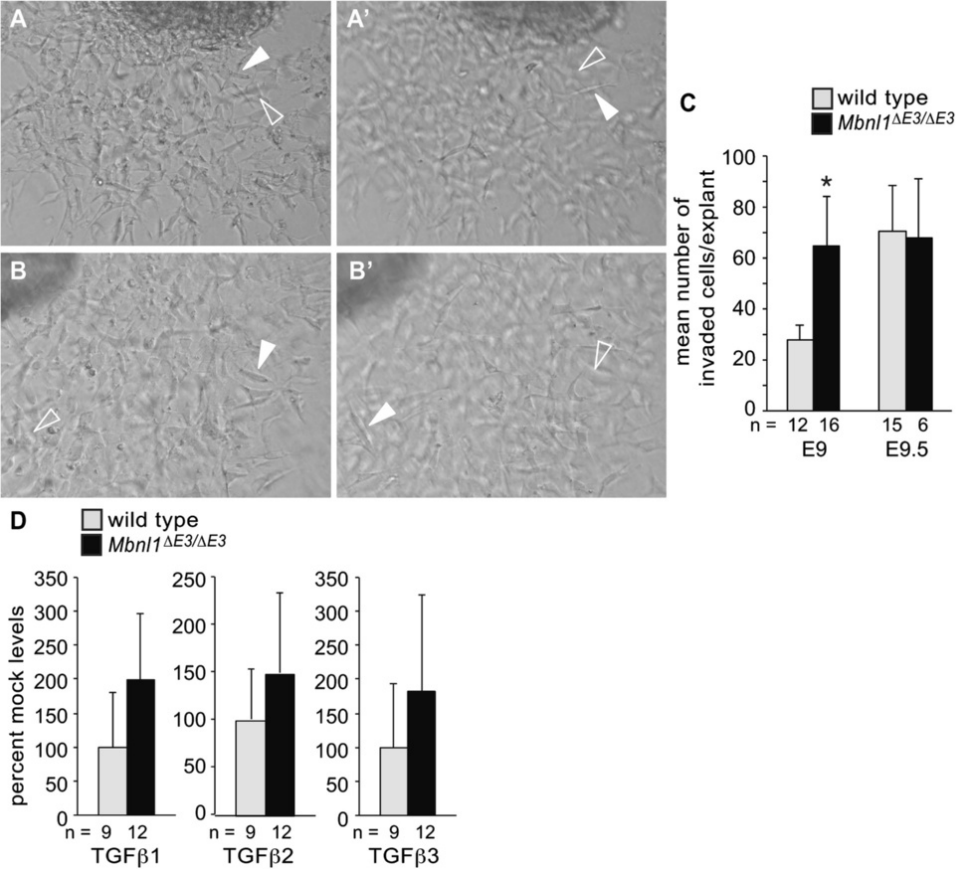
Loss of MBNL1 stimulates EMT *ex vivo* in a stage-specific manner. Images of wild type (**a**, *a*’) or *Mbnl1* ^*∆E3/∆E3*^ (**b**, *b*’) E9 AVC explant cultures were taken in the plane of focus of the surface of the gel (a, b) and beneath the surface of the gel (*a*’, *b*’) to show invaded mesenchymal cells. Filled arrowheads indicate cells in the focal plane, whereas open arrowheads indicate the same cells out of focus in a different plane. **c** AVC regions from E9 or E9.5 wild type or *Mbnl1* ^*∆E3/∆E3*^ hearts were explanted onto collagen gels. The number of explants per group is indicated. An asterisk indicates a significant difference from wild type (*P* ≤ 0.05). **d** Conditioned supernatants were collected from E9 wild type and *Mbnl1* ^*∆E3/∆E3*^ AVC explants and the levels of active TGFβ proteins secreted into the media were assayed by ELISA

We previously demonstrated that knockdown of MBNL1 in chick H&H stage 14 AVC explants promotes invasive mesenchyme production *ex vivo*, indicating that MBNL1 is a negative regulator of endocardial cushion EMT [20, 21]. To determine if this role is conserved in mammals, invasive mesenchyme production was compared in AVC explants from wild type and *Mbnl1*^*∆E3/∆E3*^ hearts (Fig. 2a-c). Indeed, E9 explants from *Mbnl1*^*∆E3/∆E3*^ mice had more invaded cells than wild type explants. In chick, the effects of MBNL1 knockdown are temporally regulated, such that knockdown of MBNL1 induces increased invasion in stage 14 AVC explants, but has no significant effect in stage 16 AVC explants [20]. Likewise, the number of invaded cells in AVC explants from E9.5 *Mbnl1*^*∆E3/∆E3*^ mice was similar to that of wild type E9.5 explants (Fig. 2c). Thus, both the stimulatory effect of loss of MBNL1 on EMT and its temporal regulation are conserved between birds and mammals.

In chick stage 14 AVC explants, elevated levels of active TGFβ3, but not TGFβ2, are secreted into the medium following MBNL1 knockdown [20]. In chick, TGFβ3 is required for invasion of mesenchymal cells, whereas TGFβ2 is required for endocardial cell activation [32]. TGFβ signaling is also important for endocardial cushion EMT in mammals, but with some differences in ligand specificity [7–9]. To determine whether the levels of active TGFβ secreted by *Mbnl1*^*∆E3/∆E3*^ AVC cushions differed from those of wild type, we collected conditioned media from wild type and *Mbnl1*^*∆E3/∆E3*^ AVC explants and measured the levels of active TGFβ1, TGFβ2, and TGFβ3 by enzyme-linked immunosorbent assay (ELISA) (Fig. 2d). There was a trend towards higher levels of all three TGFβ proteins, but differences did not reach statistical significance. This may be attributable in part to the detection limits of the assay, as the measured levels of TGFβ proteins were very low, and we failed to detect any TGFβ in some supernatants from both wild type and *Mbnl1*^*∆E3/∆E3*^ explants.

### Loss of MBNL1 leads to precocious EMT and TGFβ signaling in the endocardial cushions *in vivo* without a persistent increase in total mesenchyme production

**Fig. 3:**
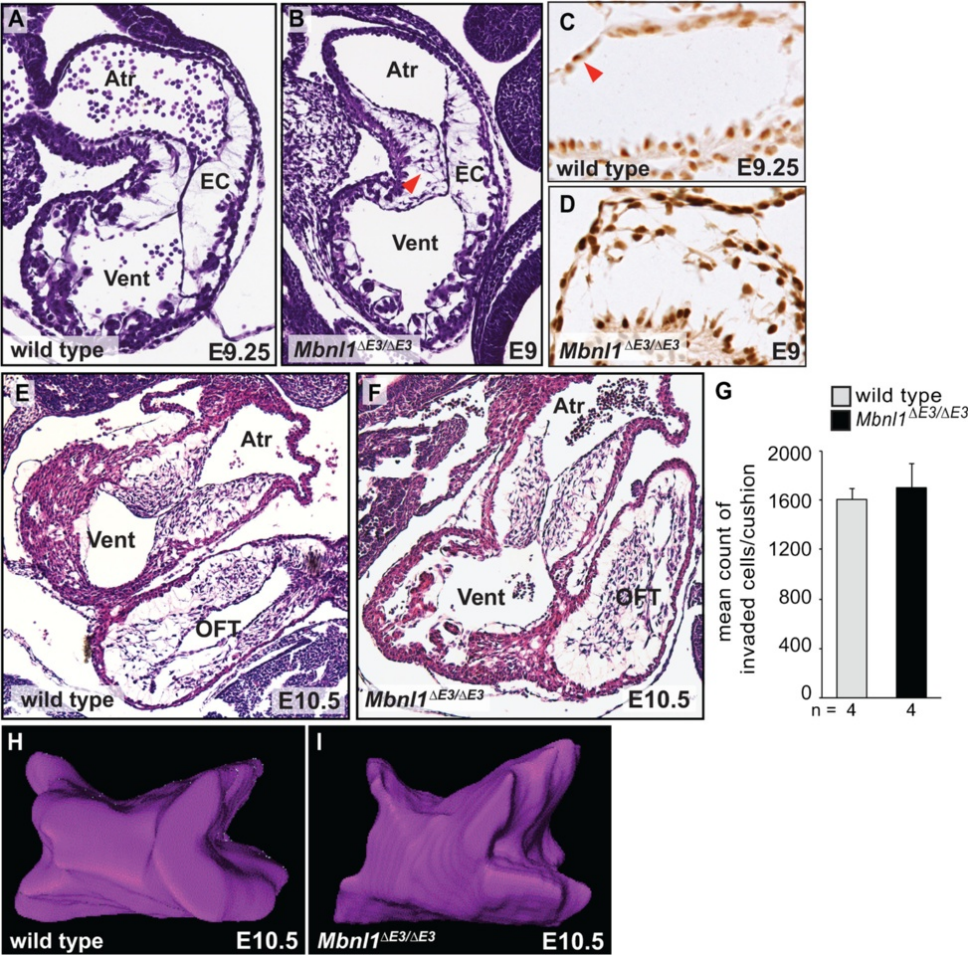
*Mbnl1* ^*E∆3/∆E3*^ mice have precocious EMT in the endocardial cushions without a persistent increase in total mesenchyme production. Close-up views of the hearts in sagittal sections of wild type E9.25 (**a**) and *Mbnl1* ^*∆E3/∆E3*^ E9 (**b**) embryos stained with H&E reveal precocious invasion of mesenchymal cells (red arrowhead) into the AVC cushions of knockout mice. Sections from three individuals were evaluated for each group; representative images are shown. Close-up views of the AVC cushions in adjacent sections from wild type E9.25 (**c**) and *Mbnl1* ^*∆E3/∆E3*^ E9 (**d**) embryos subjected to immunohistochemistry with an antibody against pSMAD2 reveal high levels of TGFβ signaling in only a few cells in wild type cushions (red arrowhead), but nearly all cells in the knockout cushions. H&E stained sagittal sections of wild type (**e**) and *Mbnl1* ^*∆E3/∆E3*^ (**f**) hearts do not reveal noticeable hypercellularity in the AVC cushions at E10.5. Atr = atrium, vent = ventricle, EC = endocardial cushion, OFT = outflow tract. **g** The average relative number of mesenchymal cells was determined by counting the cells within the AVC cushions on serial, H&E-stained, sagittal sections of wild type and *Mbnl1* ^*∆E3/∆E3*^ E10.5 embryos. Representative three-dimensional reconstructions of AVC cushions from optical sections of wild type (**h**) and *Mbnl1* ^*∆E3/∆E3*^ (**i**) E10.5 hearts imaged using optical coherence tomography. Videos of the optical sections and reconstructions of these hearts are shown in Additional file 2: Movie S1 and Additional file 3: Movie S2, respectively; still images shown here were rotated to the same viewing angle

In chick stage 14 AVC explants, knockdown of MBNL1 stimulates precocious and elevated levels of active TGFβ3 secreted into the medium, and early exposure to elevated TGFβ3 levels is sufficient to induce precocious invasion [20]. The level of invasion induced at the earlier stage by loss of MBNL1 is similar to that of control explants from the later stage in both chick and mouse [20], consistent with an accelerated induction of invasion. EMT normally begins in the mouse AVC cushions at E9.5 [33], and indeed no invaded cells were observed in heart sections from wild type embryos at E9.25 (Fig. 3a). In contrast, histological analysis of *Mbnl1*^*∆E3/∆E3*^ embryos revealed the presence of invaded cells within the AVC cushions already at E9 (Fig. 3b), suggesting that MBNL1 prevents precocious mesenchyme formation both *ex vivo* and *in vivo*. To determine whether TGFβ signaling is precociously activated in *Mbnl1*^*∆E3/∆E3*^ AVC cushions *in vivo*, we performed immunohistochemistry using an antibody against phosphorylated SMAD2 (pSMAD2), a mediator of canonical TGFβ signaling. In wild type hearts, high pSMAD2 levels were seen in a small number of AVC cushion endocardial cells at E9.25 (Fig. 3c). In *Mbnl1*^*∆E3/∆E3*^ hearts pSMAD2 was strongly detected in nearly all cells in the AVC endocardium (as well as the invaded mesenchyme) by E9 (Fig. 3d), resembling pSMAD2 staining in wild type hearts at E10 (data not shown). This indicates that the precocious production of invasive mesenchyme in the absence of MBNL1 is accompanied by precocious TGFβ signaling.

To determine whether the precocious induction of invasion leads to an over-production of cushion mesenchyme *in vivo*, the numbers of invaded cells in the AVC cushions were counted in serial sections from E10.5 wild type and *Mbnl1*^*∆E3/∆E3*^ embryos (Fig. 3e-g). Despite the early initiation of EMT in *Mbnl1*^*∆E3/∆E3*^ AVC cushions, there was no significant difference in the relative amount of AVC mesenchyme once EMT is well underway (*P* = 0.34). Imaging of E10.5 hearts using optical coherence tomography (OCT) confirmed that *Mbnl1*^*∆E3/∆E3*^ AVC cushions are indeed similar in size and shape to wild type (Fig. 3h, i and Additional file 2: Movie S1 and Additional file 3: Movie S2). Overt differences in cushion cellularity were also not observed in sections of wild type and *Mbnl1*^*∆E3/∆E3*^ hearts at E11.5, E12.5, or E14.5 (data not shown). Thus, although MBNL1 restricts the timing of EMT initiation in the AVC cushions, it is not essential for regulating the total amount of mesenchyme produced *in vivo*.

### Loss of MBNL1 has little effect on EMT in the AVC cushions at E10.5

**Fig. 4:**
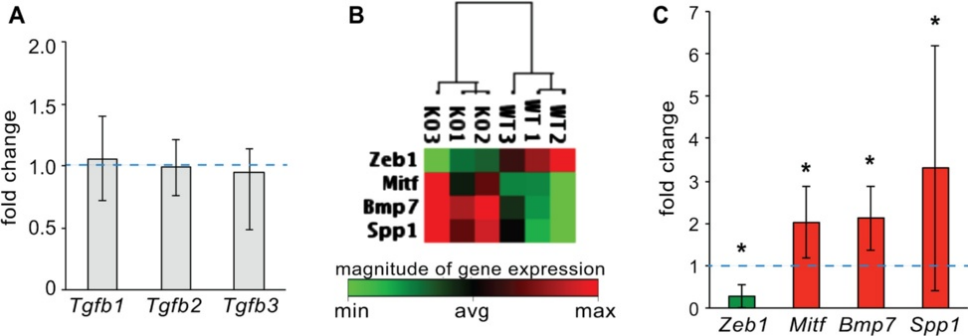
Epithelial to Mesenchymal Transition RT^2^ Profiler PCR Arrays reveal few changes in gene expression in *Mbnl1* ^*E∆3/∆E3*^ AVC cushions at E10.5. **a**-**c**) The expression of genes involved in EMT was compared in E10.5 wild type and *Mbnl1* ^*∆E3/∆E3*^ AVC endocardial cushions using the Epithelial to Mesenchymal Transition RT^2^ Profiler PCR Array (Qiagen). **a** *Tgfb1*, *Tgfb2*, and *Tgfb3* transcript levels in the knockout cushions did not differ from wild type. **b** Clustered heat map of transcripts that showed a ≥ 2 fold change in *Mbnl1* ^*∆E3/∆E3*^ cushions with P ≤ 0.05. **c** Transcript levels of the four genes that showed a ≥ 2 fold change in *Mbnl1* ^*∆E3/∆E3*^ cushions. An asterisk indicates significant difference from wild type (*P* ≤ 0.05). Error bars represent 95 % confidence intervals

At E10.5, abundant pSMAD2 staining is seen throughout the endocardium and mesenchyme of both wild type and *Mbnl1*^*∆E3/∆E3*^ AVC cushions (data not shown). To evaluate EMT-associated gene expression in the *Mbnl1*^*∆E3/∆E3*^ AVC cushions at this stage, the levels of 84 genes that either change their expression during EMT or that regulate gene expression changes during EMT were assessed by real-time RT-PCR on total RNA from wild type or *Mbnl1*^*∆E3/∆E3*^ E10.5 AVC cushions using Epithelial to Mesenchymal Transition RT^2^ Profiler PCR Arrays (Additional file 4: Table S1). TGFβ transcript levels were not affected (Fig. 4a), although it should be noted that increases in the levels of active TGFβ3 secreted by chick AVC explants following MBNL1 knockdown occurred without an accompanying increase in *TGFB3* transcript levels [20]. Consistent with equivalent amounts of mesenchyme production, both endothelial markers such as *versican* (*Vcan*) and *E-cadherin* (*Cdh1*), which are down-regulated during EMT, and mesenchymal markers such as *fibronectin* (*Fn1*) and *matrix metallopeptidase 2* (*Mmp2*), which are up-regulated during EMT, did not differ between wild type and *Mbnl1*^*∆E3/∆E3*^ cushions. Important transcriptional regulators of EMT such as *Snai1*, *Snai2* (*Slug*), and *Twist1* also do not differ between wild type and *Mbnl1*^*∆E3/∆E3*^ cushions. Only four genes (*Zeb1*, *Mitf*, *Bmp7*, and *Spp1*) differed significantly between wild type and *Mbnl1*^*∆E3/∆E3*^ cushions by at least two-fold (Fig. 4b, c). Zinc-finger E-box binding homeobox 1 (ZEB1), a transcription factor that interacts with activated SMAD proteins to promote EMT in response to TGFβ signaling [34], is down-regulated more than three-fold. Microphthalmia-associated transcription factor (MITF), which negatively regulates SMAD-dependent TGFβ signaling in mast cells [35], is up-regulated approximately two-fold. Bone morphogenetic protein 7 (*Bmp7*) levels are also doubled in *Mbnl1*^*∆E3/∆E3*^ cushions. While neither *Bmp7* nor *Bmp6* alone are essential for endocardial cushion development, double-knockout mice lacking both *Bmp7* and *Bmp6* exhibit delayed formation of the OFT cushions and defects in valve morphogenesis [36]. Finally, the glycoprotein secreted phosphoprotein 1 (*Spp1*), also known as osteopontin, is elevated more than three-fold. Transcription of *Spp1* is induced by both TGFβ and BMP signaling [37], so its up-regulation could be due to elevated TGFβ signaling or to the up-regulation of BMP7 or both. Repression of *Spp1* is required for prevent matrix mineralization in the developing heart valves [38], and elevated SPP1 levels are associated with calcific valve disease in mice and humans [39–41]. Thus, while these results suggest that TGFβ signaling is precociously activated in the *Mbnl1*^*∆E3/∆E3*^ cushions, it is unclear whether elevated TGFβ signaling is maintained once EMT is underway. Unfortunately, the small size and lack of cellularity in the cushions at E9-9.5 makes collecting enough AVC cushion tissue for a similar analysis during the initiation of EMT untenable.

### Heart valve morphogenesis is aberrant in fetal *Mbnl1*^*∆E3/∆E3*^ hearts

**Fig. 5:**
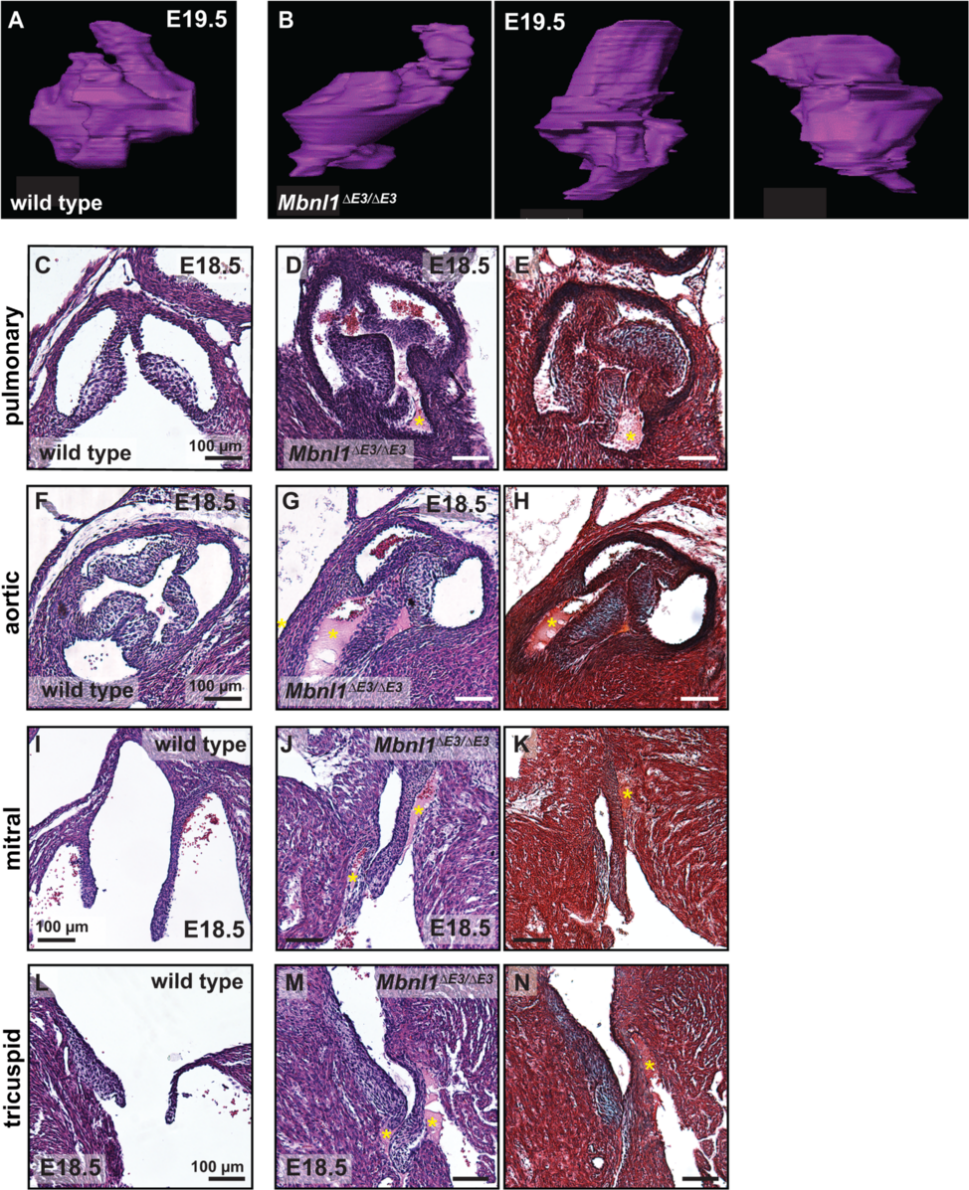
Loss of MBNL1 leads to valve dysmorphia in the fetal heart. Three-dimensional representations of the pulmonary valve at E19.5 reconstructed from optical sections of a representative wild type (**a**) and three different *Mbnl1* ^*∆E3/∆E3*^ (**b**) hearts imaged using optical coherence tomography. Videos of the optical sections and reconstructions of the wild type heart in (**a**) and the leftmost of the *Mbnl1* ^*∆E3/∆E3*^ hearts in (**b**) are shown in Additional file 5: Movie S3 and Additional file 6: Movie S4, respectively. Representative high magnification views of the pulmonary (**c**, **d**), aortic (**f**, **g**), mitral (**i**, **j**), and tricuspid (**l**, **m**) valves of E18.5 wild type (**c**, **d**, **i**, **l**) and *Mbnl1* ^*∆E3/∆E3*^ (**d**, **g**, **j**, **m**) heart sections stained with hematoxylin and eosin. Yellow asterisks indicate eosinophilic tissue connecting the valve leaflets to the vessel or chamber wall. Nearby sections through the pulmonary (**e**), aortic (**h**), mitral (**k**), and tricuspid (**n**) valves of the *Mbnl1* ^*∆E3/∆E3*^ hearts shown in (**d**, **g**, **j**, **m**) were stained with Movat’s pentachrome stain. Scale bars in panels C-N represent 100 μm

The continued expression of MBNL1 in the endocardial cushions and heart valves at post-EMT stages (Fig. 1f-h) suggests that MBNL1 may play additional roles in heart valve development beyond regulating the timing of the onset of EMT. Transformation of the endocardial cushions into the heart valves is a complex process involving expansion and condensation of the mesenchyme, differentiation of mesenchymal cells into valve interstitial cells, and changes in the composition and organization of the extracellular matrix [2, 3, 5]. Heart valve remodeling in the mouse occurs between E10.5 and E18.5. To determine whether heart valve morphogenesis is altered in *Mbnl1*^*∆E3/∆E3*^ mice, we compared the three-dimensional structures of fetal valves in E19.5 wild type and *Mbnl1*^*∆E3/∆E3*^ hearts using OCT. We chose to focus on the pulmonary valve because this valve is the most accessible at E19.5 for imaging by this method. In the E19.5 wild type heart, the pulmonary valve is globular (Fig. 5a and Additional file 5: Movie S3). Although the boundaries of the individual leaflets cannot be resolved due to their tight apposition, three distinct lobes roughly aligned in the same plane are readily appreciated. In contrast, in the E19.5 *Mbnl1*^*∆E3/∆E3*^ heart, the pulmonary valve is highly dysmorphic and its leaflets are not aligned within a single horizontal plane (Fig. 5b and Additional file 6: Movie S4).

Next, the pulmonary, aortic, mitral, and tricuspid valves were compared in sections from wild type and *Mbnl1*^*∆E3/∆E3*^ E18.5 hearts by histology (Fig. 5c-n). In four of eight *Mbnl1*^*∆E3/∆E3*^ E18.5 hearts, but none of three wild type hearts, anucleate, eosinophilic tissue connecting the valve leaflets to the vessel or chamber wall was observed in one or more valves by hematoxylin and eosin staining (Fig. 5 and data not shown). In nearby sections, this eosinophilic connective tissue stained bright red with Movat’s pentachrome stain, indicative of fibrin. Consistent with the OCT results, apparent misalignment of valve leaflets was also seen to a variable degree in *Mbnl1*^*∆E3/∆E3*^, but not wild type, hearts. Thus, in the absence of MBNL1 the remodeling of the endocardial cushions into the heart valves is aberrant in the fetal heart.

### Loss of MBNL1 results in changes in the matrix composition and TGFβ signaling in adult heart valves

**Fig. 6:**
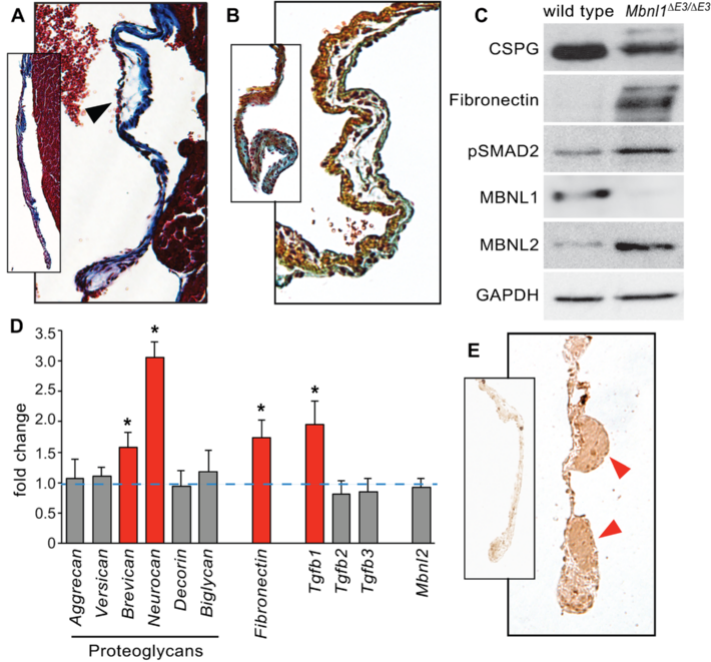
Loss of MBNL1 leads to changes in matrix composition and TGFβ signaling in the heart valves. **a** High magnification view of the septal leaflet of the tricuspid valve on a coronal section of an adult *Mbnl1* ^*∆E3/∆E3*^ heart stained with Masson’s trichrome reveals swellings of collagen-poor tissue in the valve (black arrowhead). The inset shows the corresponding valve leaflet in a wild type heart. Histological analysis was performed on sections from both male and female wild type (*n* = 7) and knockout (*n* = 12) mice ranging in age from 4 to 15 months. Representative images shown are from 5 month-old mice. **b** High magnification view of the mural leaflet of the tricuspid valve on a coronal section of an adult *Mbnl1* ^*∆E3/∆E3*^ heart stained with Movat’s pentachrome reveals normal stratification but reduced mucin staining indicative of glycosaminoglycans in the regions of swelling. The inset shows a comparable wild type leaflet. Movat’s pentachrome staining was performed on heart sections from three mice for each group ranging from 10 to 15 months old; representative images shown are from 15 month-old mice. **c** Western blot analysis confirms loss of MBNL1 protein, and shows a decrease in chondroitin sulfate proteoglycans (CSPG) and increases in fibronectin, phosphorylated SMAD2 (pSMAD2), and MBNL2 in *Mbnl1* ^*∆E3/∆E3*^ heart valves compared to wild type. Protein integrity and loading were also confirmed by Ponceau S staining (data not shown). Representatives of three independent sets using samples from 5–6 month-old male mice are shown. **d** Transcript levels in *Mbnl1* ^*∆E3/∆E3*^ heart valves were compared to those of wild type heart valves by real-time RT-PCR (*n* = 5 to 6 each, ~10 month-old males). **e** Accumulations of fibronectin (red arrowheads) were seen in the *Mbnl1* ^*∆E3/∆E3*^ tricuspid valve by immunohistochemistry. The inset shows a tricuspid valve leaflet from a wild type heart. Immunohistochemistry was performed on heart sections from three mice for each group ranging from 4 to 10 months old; representative images shown are from 4 month-old mice

Histological analysis also revealed valve dysmorphia in adult *Mbnl1*^*∆E3/∆E3*^ mice. Whereas wild type inflow valves stain predominantly blue with Masson’s trichrome stain due to their high collagen content, collagen-poor swellings sparsely populated with small, unstained, stellate cells were seen in some sections from *Mbnl1*^*∆E3/∆E3*^ hearts (Fig. 6a). Although these swellings occurred in both the mitral and tricuspid valves, they were larger and more common in the tricuspid valve. These swellings are reminiscent of those seen in periostin-null mice [42], although other defects characteristic of periostin-null valves such as loss of collagen, failure of delamination, and improper remodeling of the associated myocardium were not observed. In the mature valve, the ECM is stratified into three highly organized layers: the collagen-rich fibrosa, proteoglycan-rich spongiosa, and elastin-rich atrialis (or ventricularis in the outflow valves) [43]. Although this stratification appears relatively normal in the *Mbnl1*^*∆E3/∆E3*^ valves by Movat’s pentachrome staining, there is reduced mucin staining in the spongiosa layer in the regions of the swellings, indicative of a loss or dilution of the proteoglycans in these sites (Fig. 6b). Using an antibody against chrondroitin sulfate, lower levels of total chondroitin sulfate proteoglycan (CSPG) expression were observed in *Mbnl1*^*∆E3/∆E3*^ valves (Fig. 6c). When CSPG transcript levels were compared by real time RT-PCR, however, *aggrecan*, *versican*, *decorin*, and *biglycan* showed no difference, whereas *brevican* and *neurocan* were actually higher in *Mbnl1*^*∆E3/∆E3*^ valves (Fig. 6d). It is possible that the apparent loss of CSPGs in the *Mbnl1*^*∆E3/∆E3*^ valves is due to reduced production or attachment of the sulfated glycosaminoglycan side chains to the core proteins rather than a decline in proteoglycan expression *per se*. The ECM protein fibronectin is highly expressed in the endocardial cushions and valve precursors, but is down-regulated during maturation [44, 45]. Although *fibronectin* transcript levels did not differ between wild type and *Mbnl1*^*∆E3/∆E3*^ cushions at E10.5 (Additional file 4: Table S1), *fibronectin* transcript levels were elevated in adult *Mbnl1*^*∆E3/∆E3*^ valves by real-time RT-PCR (Fig. 6d). Elevated levels of fibronectin protein were also seen in adult *Mbnl1*^*∆E3/∆E3*^ valves by western blot (Fig. 6c), and accumulations of fibronectin were seen in the tricuspid valve by immunohistochemistry (Fig. 6e).

Western blot analyses also revealed higher levels of pSMAD2 in adult *Mbnl1*^*∆E3/∆E3*^ valves compared to wild type (Fig. 6c), indicating elevated levels of TGFβ signaling in the adult valves. Unlike precocious activation of TGFβ signaling in the endocardial cushions, this is accompanied by an up-regulation of *Tgfb1*, though not *Tgfb2* or *Tgfb3*, mRNA levels in the knockout valves (Fig. 6d).

### Loss of MBNL1 leads to valve regurgitation and atrial septal defects

To determine whether heart valve function is compromised by loss of MBNL1, transthoracic echocardiography was performed on approximately 22 week old wild type and *Mbnl1*^*∆E3/∆E3*^ mice. Color-flow and 2D-pulsed Doppler revealed a high incidence of regurgitation across the pulmonary, aortic, and mitral valves in *Mbnl1*^*∆E3/∆E3*^ mice (Table 1 and Additional file 7: Figure S2); blood flow across the tricuspid valve was not assessed. In contrast, no regurgitation was observed across the aortic or mitral valves in wild type mice. Although mild regurgitation was observed across the pulmonary valve in some wild type animals, it was less common and less severe than that observed in the knockout animals (Table 1 and data not shown).

**Table 1:** Incidence of valve regurgitation and atrial communication in wild type versus *Mbnl1* ^*∆E3/∆E3*^ hearts as assessed by echocardiography

	n^a^	%PR^a^	%AR^a^	%MR^a^	%AC^a^
Wild type males	6	83	0	0	17
Wild type females	4	25	0	0	0
Wild type, total	10	60	0	0	10
*Mbnl1* ^*∆E3/∆E3*^ males	5	80	80	80	40
*Mbnl1* ^*∆E3/∆E3*^ females	4	100	75	100	75
*Mbnl1* ^*∆E3/∆E3*^, total	9	89	78	89	56

^a^ *n* number of animals evaluated, *PR* pulmonary regurgitation, *AR* aortic valve regurgitation, *MR* mitral valve regurgitation, *AC* atrial communication

**Fig. 7:**
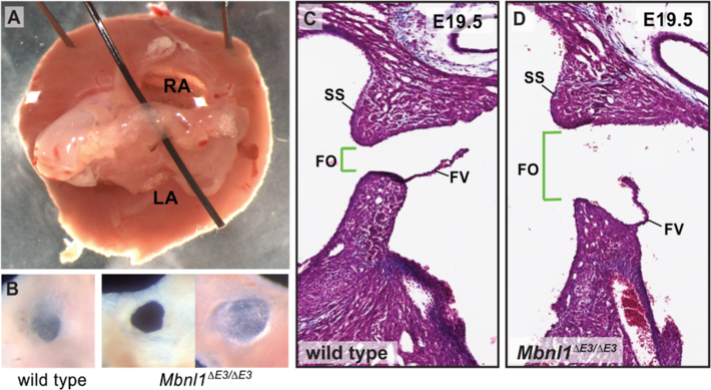
*Mbnl1* ^*E∆3/∆E3*^ mice exhibit ostium secundum atrial septal defects. **a** The heart of a one year-old adult *Mbnl1* ^*∆E3/∆E3*^ mouse is shown with the atrial appendages removed. Patency of the foramen ovale is demonstrated by the unobstructed passage of a coarse bristle. **b** Excision of the atrial septum from adult hearts shows failure of the foramen ovale to close in some *Mbnl1* ^*∆E3/∆E3*^ mice (*left*), while the foramen ovale is closed but enlarged in others (*right*). Representative images of the wild type and patent *Mbnl1* ^*∆E3/∆E3*^ foramen ovales shown are from 10 month-old mice; the image of the closed, but enlarged, foramen ovale is from a 2.5-month old *Mbnl1* ^*∆E3/∆E3*^ mouse. Compared to a wild type counterpart (**c**), the foramen ovale (bracketed by the green lines) is enlarged in an E19.5 *Mbnl1* ^*∆E3/∆E3*^ heart (**d**) as shown in close-ups of coronal sections stained with Masson’s trichrome. SS = septum secundum, FV = flap valve, FO = foramen ovale

In addition to regurgitation, atrial communication was observed in several of the *Mbnl1*^*∆E3/∆E3*^ mice, but only one of their wild type counterparts, by color and pulsed Doppler (Table 1 and Additional file 7: Figure S2). Anatomical dissection of male and female mice ranging in age from 10 weeks to one year-old confirmed the presence of a patent foramen ovale in 50 % of *Mbnl1*^*∆E3/∆E3*^ hearts, but less than 10 % of wild type hearts (Fig. 7a and Table 2). Even when successfully closed, the foramen ovale was noticeably enlarged in some *Mbnl1*^*∆E3/∆E3*^ hearts, but not in wild type (Fig. 7b and Table 2). Since atrial communication would not occur in these cases, however, there is unlikely to be any functional defect. Closure of the foramen ovale occurs in mice within the first week of postnatal life when the septum primum fuses with the septum secundum [46, 47]. When examined at late fetal stages, the foramen ovale was markedly enlarged in sections of some *Mbnl1*^*∆E3/∆E3*^ hearts (Fig. 7c-d). The infolding of the atrial roof at the superior margin (septum secundum) and the flap valve (derived from the septum primum) appear normal in these hearts, but the muscular base of the primary atrial septum where the flap valve is anchored appears reduced. Ostium secundum defects can result from either a failure to fully form the septum secundum, or from a failure of the septum primum to cover the entire oval fossa [47]. This histological analysis suggests the latter, not the former, is the reason for the high incidence of foramen ovale patency and atrial communication in the knockout mice. In other fetal *Mbnl1*^*∆E3/∆E3*^ hearts, the foramen ovale and its associated structures look similar to wild type (data not shown). No other septal defects were observed in wild type or knockout hearts.

**Table 2:** Incidence of foramen ovale defects in wild type versus *Mbnl1* ^*∆E3/∆E3*^ hearts as assessed by gross dissection

	n^a^	PFO^a^	eFO^a^
Wild type males	7	0	0
Wild type females	17	12	0
Wild type, total	24	8	0
*Mbnl1* ^*∆E3/∆E3*^ males	4	50	25
*Mbnl1* ^*∆E3/∆E3*^ females	16	50	25
*Mbnl1* ^*∆E3/∆E3*^, total	20	50	25

^**a**^ *n* number of animals evaluated, *PFO* patent foramen ovale, *eFO* enlarged, but closed, foramen ovale

Although MBNL1 is also expressed in the myocardium (Fig. 1), myocardial dysfunction has not been observed in *Mbnl1*^*∆E3/∆E3*^ mice. Lee and colleagues reported that *Mbnl1*^*∆E3/∆E3*^ hearts perform similarly to wild type in echocardiographic and electrocardiographic tests of muscle function [48]. We also evaluated myocardial function in *Mbnl1*^*∆E3/∆E3*^ mice by M-mode echocardiography of 8 week old mice (Table 3). Although slight increases in anterior and posterior wall thickness were observed during diastole, there were no differences during systole. There was a trend towards small reductions in ejection fraction and fractional shortening (to ~90 % of wild type values), but these did not reach statistical significance, consistent with previous reports [48]. Total heart size, measured either as a percentage of total body weight or as heart weight normalized to tibia length, also did not differ between wild type and *Mbnl1*^*∆E3/∆E3*^ mice at four months of age (Table 4).

**Table 3:** Cardiac function as assessed by M-mode echocardiography in wild type versus *Mbnl1* ^*∆E3/∆E3*^ hearts

	Wild type male (*n* = 8)	*Mbnl1* ^*∆E3/∆E3*^ male (*n* = 7)	*P* value	Wild type female (*n* = 5)	*Mbnl1* ^*∆E3/∆E3*^ female (*n* = 7)	*P* value
Heart rate (beats/min)	491.6 ± 21.7	512.7 ± 22.2	0.25	482.2 ± 38.7	488.7 ± 21.2	0.44
Anterior wall thickness, diastole (mm)	0.43 ± 0.02	0.48 ± 0.02	0.04*	0.43 ± 0.02	0.49 ± 0.03	0.03*
Anterior wall thickness, systole (mm)	0.57 ± 0.03	0.60 ± 0.02	0.18	0.53 ± 0.03	0.54 ± 0.02	0.40
LV diameter, diastole (mm)	3.96 ± 0.16	4.17 ± 0.16	0.18	3.79 ± 0.10	3.70 ± 0.06	0.25
LV diameter, systole (mm)	2.43 ± 0.13	2.75 ± 0.22	0.12	2.31 ± 0.15	2.38 ± 0.06	0.33
LV posterior wall thickness, diastole (mm)	0.52 ± 0.02	0.52 ± 0.03	0.49	0.46 ± 0.02	0.55 ± 0.03	0.03*
LV posterior wall thickness, systole (mm)	0.56 ± 0.02	0.58 ± 0.04	0.31	0.53 ± 0.01	0.55 ± 0.01	0.19
Ejection fraction (%)	75.3 ± 1.8	69.4 ± 4.1	0.11	75.8 ± 2.8	72.1 ± 1.2	0.14
Fractional shortening (%)	38.6 ± 1.6	34.7 ± 3.4	0.16	39.4 ± 2.4	35.9 ± 1.0	0.11

* Significant difference (P < 0.05) from wild type

**Table 4:** Heart size in wild type versus *Mbnl1* ^*∆E3/∆E3*^ mice

	Wild type male (*n* = 15)	*Mbnl1* ^*∆E3/∆E3*^ male (*n* = 12)	*P* value	Wild type female (*n* = 11)	*Mbnl1* ^*∆E3/∆E3*^ female (*n* = 9)	*P* value
Percentage of body weight	0.52 ± 0.02	0.53 ± 0.04	0.39	0.46 ± 0.02	0.51 ± 0.03	0.10
Heart weight/tibia length (mg/mm)	9.72 ± 0.49	9.13 ± 0.59	0.22	6.44 ± 0.12	7.12 ± 0.42	0.07

It has previously been reported that the closely related paralog, MBNL2, can compensate for loss of MBNL1 in the myocardium. MBNL2 protein levels are elevated in the hearts of *Mbnl1*^*∆E3/∆E3*^ mice [48]. MBNL1 and MBNL2 recognize similar RNA elements [49, 50], and MBNL2 binding to MBNL targets increases when MBNL1 is absent [48]. Restoring MBNL2 to near wild type levels in *Mbnl1*-null mice by removal of one *Mbnl2* allele was sufficient to induce atrial dilation, ventricular hypertrophy, and conduction defects [48]. In these compound knockout mice MBNL2 levels were assessed in whole heart, however, leaving it unclear whether MBNL2 was elevated specifically within the myocardium, which comprises the majority of the heart by volume, or in all cells that normally express MBNL1. We tested whether loss of MBNL1 results in increased MBNL2 levels within the heart valves by western blot and real-time RT-PCR (Fig. 6c, d). We found that MBNL2 protein levels were higher in *Mbnl1*^*∆E3/∆E3*^ valves, although *Mbnl2* transcript levels remained unchanged, suggesting post-transcriptional regulation at the level of translation or protein stability. Thus, the up-regulation of MBNL2 is not restricted to muscle tissue. The presence of valve dysmorphia and regurgitation in *Mbnl1*^*∆E3/∆E3*^ mice, however, indicates that unlike in the myocardium, higher levels of MBNL2 are insufficient to fully compensate for loss of MBNL1 in the heart valves.

### Pigmentation is increased in *Mbnl1*^*∆E3/∆E3*^ heart valves

**Fig. 8:**
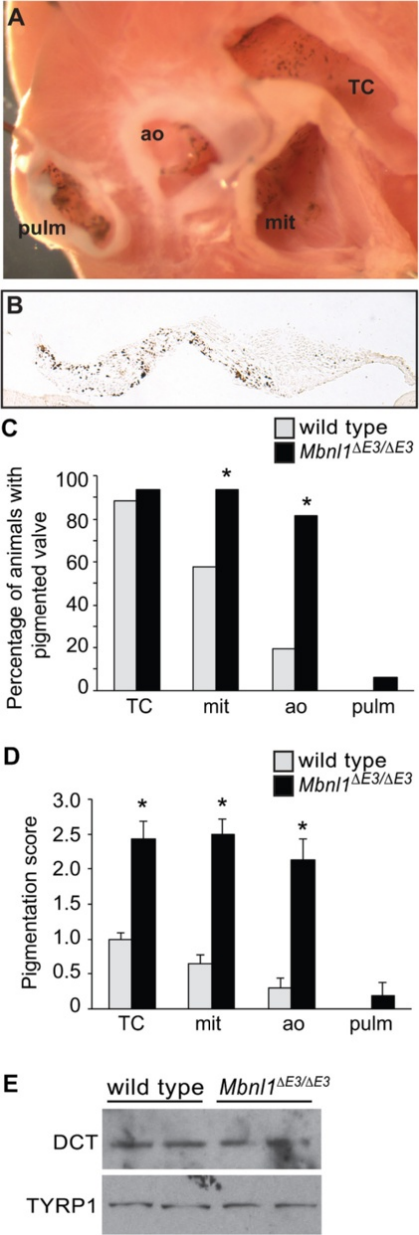
*Mbnl1* ^*∆E3/∆E3*^ heart valves exhibit increased valve pigmentation. **a** A top view of a 10 month-old adult *Mbnl1* ^*∆E3/∆E3*^ heart with the major vessels and atrial appendages removed is shown. Pigment can be seen in all four valves of this heart. TC = tricuspid valve, mit = mitral valve, ao = aortic valve, pulm = pulmonary valve. **b** The accumulation of pigment is readily apparent in a representative high magnification view of the mitral valve on an unstained coronal section of a 10 month-old adult *Mbnl1* ^*∆E3/∆E3*^ heart. **c** The percentage of each valve with visible pigment was calculated for wild type (*n* = 26) and *Mbnl1* ^*∆E3/∆E3*^ (*n* = 16) hearts from male and female mice ranging from 2.5 to 10 months old. No differences were observed between the sexes. **d** The extent of pigmentation was compared for each valve using a scoring system of 0 (no melanin) to 3 (abundant melanin) in the hearts from (**c**); see Additional file 8: Figure S3 for examples. An asterisk indicates a significant difference from wild type (P ≤ 0.05). **e** Western blots analysis shows no difference in the expression of melanocyte markers in wild type versus *Mbnl1* ^*∆E3/∆E3*^ heart valves. Each lane represents a pool of all four valves (tricuspid, mitral, aortic, and pulmonary) from one individual. A representative of two blots (*n* = 2 or 3 independent samples on each blot from 5–6 month-old males) is shown. Protein integrity and equivalent loading were confirmed by Ponceau S staining (data not shown)

During dissection and collection of heart valves from adult wild type and *Mbnl1*^*∆E3/∆E3*^ hearts, we observed a greater amount of pigment in valves from the knockout mice (Fig. 8). The presence of cardiac melanocytes in murine heart valves has been well documented [51–56]. Lineage tracing studies have demonstrated that these melanocytes are derived from invading neural crest cells, and normally populate the tricuspid, mitral, and aortic valves, but not the pulmonary valve [53, 55]. We saw an increase in the presence of pigmentation in both inflow and outflow valves in *Mbnl1*^*∆E3/∆E3*^ hearts, including one instance of pigment in the pulmonary valve (Fig. 8a-c). In order to compare the amount of pigment in wild type and *Mbnl1*^*∆E3/∆E3*^ valves, we developed a pigmentation scoring system (Additional file 8: Figure S3) in which each valve was given a score of 0 (no melanin) to 3 (abundant melanin). In addition to an increased incidence of pigmentation, we also saw an increase in the amount of pigment within pigmented valves (Fig. 8d). The level of pigmentation has been shown to correlate with coat color in mice [55]. All of our wild type and *Mbnl1*^*∆E3/∆E3*^ mice are on the B6129 background, a hybrid of C57BL/6 black mice and 129 albino mice, and have either brown or black coats. The incidence and relative amount of pigmentation in wild type or *Mbnl1*^*∆E3/∆E3*^ valves were the same regardless of coat color (data not shown), so data from brown and black mice were pooled in the graphs in Fig. 8. Despite these differences in pigmentation, wild type and *Mbnl1*^*∆E3/∆E3*^ valves express similar levels of dopachrome tautomerase (DCT), a marker of the melanoblast lineage found in melanocytes and their precursors, and tyrosinase related protein 1 (TYRP1), a marker of differentiated melanocytes (Fig. 8e).

## Discussion

The present study demonstrates that MBNL1 is required for normal heart valve development and function. *Mbnl1*^*∆E3/∆E3*^ mice, which lack MBNL1 protein, have valve dysmorphia, alterations in ECM composition, and regurgitation across both inflow and outflow valves. Loss of MBNL function has been implicated in the pathogenesis of myotonic dystrophy (*dystrophia myotonica*, DM), a multisystemic genetic disorder characterized by a number of cardiac, muscle, endocrine, and neurological defects [57]. DM is caused by the expression of mutant RNAs containing expanded CUG (DM1) or CCUG (DM2) repeats, which leads to the dysregulation of other transcripts in part through the sequestration of MBNL proteins (for a detailed review of DM pathogenesis see [57]). Notably, DM patients exhibit a variety of cardiac abnormalities, including a high incidence of mitral valve prolapse [58]. *Mbnl1*^*∆E3/∆E3*^ mice recapitulate many of the features of DM, including skeletal muscle myotonia and cataracts [59], but a valve phenotype had not previously been described. Valve dysfunction in DM patients has generally been assumed to be a consequence of myopathy, caused by weakness of the papillary muscles to which the inflow valves connect [58]. Although *Mbnl1*^*∆E3/∆E3*^ mice do not exhibit overt cardiomyopathy, loss of MBNL1 function in the papillary muscles could predispose the mice to tricuspid and mitral valve failure. This would not explain differences in function of the aortic and pulmonary valves in *Mbnl1*^*∆E3/∆E3*^ mice, however, which are free standing. The expression of both MBNL1 and MBNL2 in wild type adult heart valves argues instead for a direct function of MBNL proteins in heart valve homeostasis. Suggestively, *MBNL2* is one of sixteen genes within a locus on chromosome 13 that has been genetically linked with mitral valve prolapse [60], although to date no studies have corroborated a role for MBNL2 in valve disease. Both nuclear and cytoplasmic roles of MBNL proteins have been implicated in DM pathogenesis, including regulation of pre-mRNA alternative splicing, alternative polyadenylation, microRNA biogenesis, mRNA localization and stability [22, 24, 25, 29]. It remains to be determined which of these roles are essential in the developing valves.

We previously showed that knockdown of MBNL1 in chicken endocardial cushion explants enhanced autocrine TGFβ3 and invasive mesenchyme production *ex vivo* in a stage-dependent manner [20, 21]. Here, we demonstrate that loss of MBNL1 has a similar effect in mouse endocardial cushion explants *ex vivo*. In chick explants, activation (i.e., separation of cells from the endothelial monolayer) and invasion (migration into the collagen gel) are easily distinguishable steps of EMT; unfortunately, in mouse endocardial cushion explants activation is not readily apparent, as cells appear to invade directly from the explant into the collagen gel [13]. Thus, although we saw a stage-dependent increase in the number of invaded cells in *Mbnl1*^*∆E3/∆E3*^ explants, we cannot say whether invasion *per se* is specifically stimulated as we observed in the chick [20, 21]. Loss of MBNL1 stimulates precocious TGFβ signaling and EMT in mouse endocardial cushions *in vivo*, which is also consistent with our previous finding that knockdown of MBNL1 in chick endocardial cushion explants stimulates precocious secretion of active TGFβ3 *in vitro* [20]. This effect is temporary in the mice, however, as the amount of total mesenchyme produced is normalized by E10.5, and few changes in EMT gene expression are observed at this stage. Taken together, these data suggest that MBNL1 regulates the initiation of EMT, but not its progression. The dysmorphia and presence of abnormal connective tissue in the fetal valves indicates a developmental role for MBNL1 in valvulogenesis; if not regulating the amount of cushion mesenchyme, then perhaps influencing its fate during valve maturation.

The stratification of the adult valve suggests that matrix remodeling is relatively unimpaired in *Mbnl1*^*∆E3/∆E3*^ mice. The presence of collagen/proteoglycan-poor swellings sparsely populated with stellate cells, however, indicates localized increases in matrix with an atypical composition. Indeed, while CSPGs levels are reduced, the ECM protein fibronectin is strongly up-regulated. The maintenance of stratification and the specific changes in ECM composition observed in the adult *Mbnl1*^*∆E3/∆E3*^ valves are not characteristic of either myxomatous valve degeneration or calcific valve disease. The persistence of fibronectin in adult *Mbnl1*^*∆E3/∆E3*^ valves could be indicative of incomplete or defective differentiation of the cushion mesenchyme in the knockout mice. MBNL1 has recently been shown to regulate alternative splicing changes that are important for stem cell differentiation [61, 62], and MBNL1 has been implicated in the differentiation of skeletal muscle, blood, lens, and adipose cells [63–67]. Alternatively, elevated fibronectin expression in the adult valves could be indicative of injury to the valves, perhaps due to changes in shear stress caused by altered valve biomechanics. Valve interstitial cells have been shown to up-regulate fibronectin in response to injury in primary cultures [68]. The accumulation of fibronectin in the adult valve may in turn then have a detrimental effect on valve function by altering the valve’s elasticity or stiffness.

The higher levels of fibronectin in adult *Mbnl1*^*∆E3/∆E3*^ valves could also potentially have a protective effect. We found that both *Tgfb1* transcript levels and TGFβ signaling (as indicated by pSMAD2) are elevated in the adult knockout valves. Elevated TGFβ1 levels are associated with calcification in stenotic valves, and addition of TGFβ1 is sufficient to induce calcification in valve interstitial cells in culture [69]. We have observed no signs of overt calcification in *Mbnl1*^*∆E3/∆E3*^ valves, however, even in animals as old as 90 weeks (data not shown). Notably, fibronectin has been shown to protect cultured valve interstitial cells from TGFβ1-induced calcification [70]. The mechanisms by which TGFβ1 and fibronectin are up-regulated in adult *Mbnl1*^*∆E3/∆E3*^ valves, and the extent to which MBNL1 exerts its effects in the mature valve through modulation of the ECM and/or TGFβ activity, remain to be determined.

The relevance of increased pigmentation in the *Mbnl1*^*∆E3/∆E3*^ heart valves is unclear. Pigmented cells were first observed in the mouse heart over fifty years ago [54]. Although gene expression profiling suggests these cardiac melanocytes are more similar to cardiomyocytes than canonical melanocytes found in the skin, these neural crest-derived cells express melanocyte markers and produce melanin in non-albino mice [51–53, 55, 56]. DCT-expressing cells have also been reported in human heart valves, although these cells lack TYRP1 and are not pigmented [56]. The purpose of these cells is not well understood. One suggestion has been that the enzymes within the melanin synthesis pathway buffer calcium and reactive species in the heart [56]. Melanin is synthesized from the amino acid tyrosine in a multistep process in which DCT converts the intermediate dopachrome into 5,6-dihydroxyindole carboxylic acid (DHICA), which is then converted into eumelanin by TYRP1 [71]. The normal levels of DCT and TYRP1 in the *Mbnl1*^*∆E3/∆E3*^ valves suggests that the increase in pigmentation is not due to an increase in the number of melanocytes in the valves, or an up-regulation in these steps of melanin production. Little is known about melanin turnover. The accumulation of pigment in the heart valves is associated with stiffening of the valves, regurgitation, and stenosis in patients with alkaptonuria, a rare autosomal recessive disorder of tyrosine metabolism characterized by ochronosis, the accumulation of pigment within the connective tissues [72–75]. An *ex vivo* nanomechanical study of mouse tricuspid valves reported that pigmented regions of the tricuspid leaflet are stiffer than regions lacking pigment [76]. Thus, increased pigment in the valves of *Mbnl1*^*∆E3/∆E3*^ mice could contribute to their functional impairment by altering their biomechanical properties.

The impact of loss of MBNL1 in the heart is not confined to the developing valves. Many *Mbnl1*^*∆E3/∆E3*^ mice had ostium secundum defects. A strong association between ostium secundum defects and mitral valve prolapse has been documented [77, 78]. Examination of the fetal heart prior to closure revealed that the septum secundum, which forms from infolding of the atrial roof at the superior margin, and the septum primum, which forms the flap valve, appear normal in the knockout mice. The muscular base to which the flap valve is anchored, however, is smaller in some *Mbnl1*^*∆E3/∆E3*^ hearts. The muscular base of the atrial septum arises from myocardialization of mesenchyme within the dorsal mesenchymal protrusion (DMP), a derivative of the secondary heart field, not the endocardial cushions [47]. Underdevelopment of the DMP can be caused by reduced proliferation, increased apoptosis, or premature myocardialization of the DMP mesenchyme [47]. Mutations in several genes, including cardiac transcription factors such as *NKX2.5*, *GATA4*, and *TBX20*, have been linked to atrial septal defects and foramen ovale patency in humans [79–81], but their precise roles in the development and maturation of the DMP are not known. Often, defects in the DMP are associated with more severe atrioventricular septal defects, but these, or other types of septal defects that arise from defective remodeling of the endocardial cushions, are not observed in *Mbnl1*^*∆E3/∆E3*^ mice. Although atrial communication can lead to right atrial overload and other cardiac complications, stand-alone ostium secundum defects are often clinically benign [47].

The lack of overt cardiomyopathy in *Mbnl1*^*∆E3/∆E3*^ mice despite both atrial communication and poor valve function might initially be surprising, but it should be noted that these mice also experience progressive skeletal muscle myotonia [59]. As *Mbnl1*^*∆E3/∆E3*^ mice age and their myotonia worsens, their level of activity decreases, which may help reduce their cardiac burden. The up-regulation of MBNL2 may also help the strained heart muscle compensate [48]. It has also been reported, however, that *Mbnl1-*knockout mice on a pure 129 sv genetic background (as opposed to *Mbnl1*^*∆E3/∆E3*^ mice, which are on a hybrid background of C57/BL6 and129 strains) exhibit progressive cardiac dysfunction, cardiac hypertrophy, and myocardial cell death and fibrosis despite a similar up-regulation of MBNL2 [82]. The difference in heart muscle pathogenesis between the mouse strains suggests the existence of modifier genes that modulate the function of MBNL1 in the heart. MBNL1-dependent heart valve phenotypes have not been investigated on a pure 129 background.

## Conclusions

MBNL1 is highly expressed in the endocardial cushions and heart valves. It plays a conserved role in regulating the onset of TGFβ signaling and EMT in the endocardial cushions, but is not essential for limiting the total amount of cushion mesenchyme production *in vivo*. Mice lacking MBNL1 exhibit defects in valve morphogenesis, ECM composition, function, and pigmentation, indicating that MBNL1 is required for normal post-EMT valve development.

## Methods

### *Mbnl1*^*∆E3/∆E3*^ Mice

Mouse studies were conducted in strict accordance with the recommendations of the American Veterinary Medical Association and under the approval of the Cleveland Clinic Institutional Animal Care and Use Committee (Protocols 2011–0547 and 2014–1201). All efforts were made to minimize pain and distress during mouse animal husbandry and euthanasia. *Mbnl1*^*∆E3/∆E3*^ mice have a targeted deletion of *Mbnl1* exon 3, which contains the canonical translation start codon, resulting in loss of MBNL1 expression [59]. Mice were maintained in a B6129 hybrid background. Embryos at specific stages were obtained by timed mating. In general, midday following observation of a copulation plug was considered embryonic day 0.5 (E0.5). Stages of embryos collected from E9 through E10.5 were confirmed by counting the number of somites of each individual embryo (E9 = 19–20 somites, E9.5 = 22–23 somites, E10.5 = 34–36 somites).

### Immunohistochemistry and western blotting

For immunohistochemistry, whole embryos or postnatal hearts were fixed overnight by immersion in 4 % paraformaldehyde (embryonic stages) or 10 % neutrally buffered formalin (adult), and then embedded in paraffin. Immunohistochemistry was performed on 10 μm sections. The sections were immersed in xylene, rehydrated through a 100 %, 95 %, and 80 % ethanol series, and then washed in water. Antigen retrieval was performed by boiling slides in citrate buffer (10 mM citric acid, 0.05 % Tween 20, at pH 6.0) for 30 min. The slides were washed three times in 1x Tris-buffered saline (TBS) and blocked in 3 % goat serum in 1x TBS for 1 h at room temperature. The primary antibody was added at a 1:50 to 1:400 dilution and incubated overnight at 4 °C. The slides were washed in TBS as before, incubated in 0.3 % hydrogen peroxide for 30 min at room temperature, and washed in 1x TBS. The secondary antibody was added at a 1:400 dilution and incubated for one hour at room temperature. Slides were washed 3 times in 1X TBS and developed using DAB Plus Substrate Kit (Invitrogen) according to the manufacturer’s instructions. The slides were dehydrated through a 25 %, 50 %, 75 %, and 100 % ethanol series, cleared with xylene, and mounted with Permount (Fisher Scientific). Negative controls lacking primary antibody were performed in parallel for every experiment.

Heart tissues were homogenized in protein loading buffer and subjected to western blotting as previously described [30]. Protein integrity and equivalent loading were verified by Ponceau S staining. For western blots on adult heart valves, all four valves (tricuspid, mitral, aortic, and pulmonary) from one to three individuals were pooled together for each sample. All westerns were performed on three or more independent pooled samples from each group.

Primary antibodies used for immunohistochemistry and western blotting were: MBNL1 = rabbit polyclonal anti-MBNL1 (Aviva Systems Biology, catalog number ARP41227_P050) for immunohistochemistry, and mouse monoclonal anti-MBNL1, 3A4 [83] or rabbit polyclonal anti-MBNL1, A2764 (generously provided by Charles A. Thornton, University of Rochester, NY) for western blotting; pSMAD2 = rabbit polyclonal anti-phospho-Smad2 (Ser465/467) (Cell Signaling Technology, catalog number 3101); CSPG = mouse monoclonal anti-chondroitin sulfate, CS-56 (Sigma, catalog number C8035); Fibronectin = rabbit polyclonal anti-fibronectin (Sigma, catalog number F3648); GAPDH = mouse monoclonal anti-GAPDH, 6G5 (Biogenesis, catalog number 4699–9555); DCT = goat polyclonal anti-TRP2, D-18 (Santa Cruz, catalog number sc-10451); TYRP1 = rabbit polyclonal anti-TRP1, H-90 (Santa Cruz, catalog number sc-25543); MBNL2 = mouse monoclonal anti-MBNL2, 3B4 (Santa Cruz, catalog number sc-136167). Secondary antibodies used were: goat anti-rabbit-HRP (Calbiochem, catalog number 401393); goat anti-mouse-HRP (Calbiochem, catalog number DC02L); donkey anti-goat-HRP (Santa Cruz, sc-2056).

### Explants

Collagen gels were prepared as previously described [21]. Collagen gels were incubated in Hydration Medium [Opti-MEM supplemented with 1 % each ITS (Invitrogen), fetal bovine serum, and pen/strep antibiotic solution] for 30 min at 37 °C prior to use, which was then removed before explants were placed on the gel. The AVC regions were excised from E9 or E9.5 embryos, cut open, placed endothelial-side down on the collagen gels, and incubated at 37 °C in 5 % CO_2_. After 12 h, Mouse M199+ [M199 supplemented with 1 % each ITS (Invitrogen), fetal bovine serum, and pen/strep antibiotic solution] was added to each well. Explants were fixed after 48 h of culture in 4 % paraformaldehyde for 45 min at room temperature, and stored in PBS at 4 °C. Invaded cells were counted on a Leica DMIRB microscope fitted with Hoffman Modulation Contrast optics. Explants were imaged using QCapture Pro 6.0 software.

For enzyme-linked immunosorbent assay (ELISA) experiments, 200 μl of Mouse M199+ medium was added to each explant at 12 h, and the conditioned medium was collected at 48 h. Secreted active TGFβ levels in the conditioned media were measured by sandwich ELISA as previously described [20]. A pan-TGFβ antibody (R&D Systems, catalog number MAB1835) was used for capture, and a specific biotinylated anti-TGFβ1 (R&D Systems, catalog number BAF240), anti-TGFβ2 (R&D Systems, catalog number BAF302), or anti-TGFβ3 (R&D Systems, catalog number BAF243) was used for detection. Serial dilutions of recombinant TGFβ1, TGFβ2, and TGFβ3 (R&D Systems, catalog numbers 240-B-002, 302-B2, and 243-B3, respectively) were used to generate standard curves.

### Gene expression and alternative splicing

Total RNA was extracted using Trizol Reagent (Thermo Scientific) and quantified using a NanoDrop 1000. Endocardial cushions were dissected out of wild type and *Mbnl1*^*∆E3/∆E3*^ E10.5 embryos by cutting open the heart tube and separating the cushions from the underlying myocardium using an electrolytically-sharpened tungsten needle [84]. Both the superior and inferior AVC cushions were collected from each embryo, and the cushions from 10–14 embryos (one to two litters) were combined and treated as a single biological replicate. Three such pooled samples were collected for each group. The expression levels of 84 genes that either change their expression during EMT or regulate gene expression changes during EMT were assessed by real-time RT-PCR using the Epithelial to Mesenchymal Transition RT^2^ Profiler PCR Array (Qiagen) according to the manufacturer’s instructions. Transcript levels were normalized to *Gusb*; similar results were obtained when data were normalized against *Gapdh* (data not shown). Error bars represent 95 % confidence intervals.

For gene expression changes in adult heart valves, each sample represents a pool of all four heart valves (tricuspid, mitral, aortic, and pulmonary) from a single individual. Transcript levels were determined by real-time RT-PCR using SYBR Green Master Mix (Applied Biosystems) and normalized to *Gapdh* as previously described [85]. Primer sequences for *aggrecan, versican, brevican, neurocan, decorin, bigylcan, and scleraxis* were taken from Barnette et al. [86]; sequences of the remaining primers are shown in Table 5. Samples from five to six individuals per group were each run in triplicate. Error bars represent standard error of the mean values for the biological replicates.

**Table 5:** qRT-PCR primer sequences used in this study

Gene	Forward (F) and Reverse (R) primer sequences	Product size (bp)
*Fibronectin*	F: CAGATGCAACGATCAGGACAC	272
	R: CAGATCCGGCTGAAGCACTTT	
*Tgfb1*	F: ATACGCCTGAGTGGCTGTCT	123
	R: AGTGAGCGCTGAATCGAAAG	
*Tgfb2*	F: CTGGAACCACTGACCATTCTC	131
	R: CGTGATTTTCGTGTCCTGGC	
*Tgfb3*	F: TGAATGGCTGTCTTTCGATG	102
	R: GTGACATGGACAGTGGATGC	
*Mbnl2*	F: CCCCGTGAGAGACACAAAGTG	235
	R:CCTCCCATTAATCTCTAGCTGG	
*Gapdh*	F: TCGTCCCGTAGACAAAATGG	132
	R: TTGAGGTCAATGAAGGGGTC	

### Histology

Whole embryos or postnatal hearts were fixed overnight by immersion in 4 % paraformaldehyde (embryonic stages) or 10 % neutrally buffered formalin (adult). Fixed embryos or hearts were embedded in paraffin, sectioned, and stained with hematoxylin and eosin (H&E), Masson’s trichrome, or Movat’s pentachrome stain. To determine the relative number of mesenchymal cells at E10.5, 10 μm sections were taken through the entire heart, those sections containing AVC cushions were identified, and six sections taken 10 μm apart (every other section) from the center of the AVC region were stained with H&E, imaged on a Leica DM 2500 microscope using QCapture Pro 6.0 software, and counted. Invaded cells were counted in the cushions of four embryos per group.

### Optical coherence tomography (OCT)

Wild type and *Mbnl1*^*∆E3/∆E3*^ embryos were collected at E10.5 (*n* = 10 and 6, respectively) or E19.5 (*n* = 3 each), fixed overnight in 4 % paraformaldehyde, dehydrated through a 25, 50, 75, 100 % methanol series in PBSw (PBS with 0.1 % Tween-20), and stored in 100 % methanol at −20 °C. Embryos were later rehydrated through a 100, 75, 50, 25 % methanol series in PBSw followed by transfer into PBS. The hearts were removed under a dissecting microscope. E19.5 hearts were cleared prior to imaging using the *Clear*^*T*^ method described for whole dissected brains (E16-P11) [87], which has also been used for embryonic day 8 quail embryo hearts [88], to improve penetration depth. Embryos were imaged using a custom-built Fourier domain OCT system with a quasi-telecentric scanner, linear-in-wavenumber spectrometer and a line-scan camera with a line rate of 47 kHz [89, 90]. The axial resolution as well as the lateral resolution is approximately 10 μm in tissue. Thus OCT imaging offers high spatial resolution and good penetration depth (1–3 mm in cardiac tissues) for phenotyping embryonic hearts [91]. Custom MATLAB programs (MathWorks; Natick, MA) were utilized to create OCT images from the raw data. Amira software (FEI Visualization Sciences Group; Burlington, MA) was used to visualize OCT data.

### Heart function and size

Cardiac structural and functional changes were assessed *in vivo* by echocardiography performed on age- and sex-matched wild type and *Mbnl1*^*∆E3/∆E3*^ mice anesthetized with isofluorane gas (1-2 % with 2 lpm oxygen) using a GE Vivid7 Ultrasound system equipped with a 14 MHz iL13 probe. Two-dimensional and M-mode echocardiographic measurements of the anterior wall, posterior wall, and left ventricular diameter were recorded in the parasternal short axis view of the left ventricle at the level of the papillary muscles. Modified short-axis views were recorded to assess the possibility of atrial or ventricular septal defects, and to obtain pulmonary, aortic, and mitral valve Doppler flow measurements. Functional parameters were calculated using standard formulas [92]. To control for differences in body size, heart size was evaluated both as the percentage of total body weight that is heart weight and as a ratio of heart weight to tibia length.

### Statistics

Data are reported as the mean + standard error of the mean unless otherwise noted. Comparisons between means were performed via *t*-test assuming unequal variances. Comparisons between proportions were performed via *z*-test. Differences were considered statistically significant when P ≤ 0.05.

## Additional files

Additional file 1: Figure S1. MBNL1 expression in the embryonic brain. Immunohistochemistry with an anti-MBNL1 antibody was performed on sagittal sections from E9 wild type mouse embryos. Close-up of the head shows strong MBNL1 expression in the ectoderm lining the entrance to Rathke’s pouch (Rp), forebrain (FB), midbrain (MB), and hindbrain (HB) with little to no detectable staining within the interior encephalic tissues (highlighted by asterisks). A representative section from one of four embryos is shown. (PDF 1247 kb) Additional file 2: Movie S1. Imaging of the AVC cushions in a representative wild type heart at E10.5 by OCT. Optical sections through a representative E10.5 wild type heart are shown. The AVC cushions are highlighted in purple, and a three-dimensional reconstruction of the AVC cushions is shown. A still image of this 3D reconstruction is shown in Fig. 3h, but has been rotated and scaled to match the orientation and magnification of the *Mbnl1* ^*∆E3/∆E3*^ image in Fig. 3i. (MOV 2333 kb) Additional file 3: Movie S2. Imaging of the AVC cushions in a representative *Mbnl1* ^*∆E3/∆E3*^ heart at E10.5 by OCT. Optical sections through a representative E10.5 *Mbnl1* ^*∆E3/∆E3*^ heart are shown. The AVC cushions are highlighted in purple, and a three-dimensional reconstruction of the AVC cushions is shown. A still image of this 3D reconstruction is shown in Fig. 3i. (MOV 1197 kb) Additional file 4: Table S1. Epithelial to Mesenchymal Transition RT^2^ Profiler PCR Array data for *Mbnl1* ^*∆E3/∆E3*^ versus wild type E10.5 AVC cushions. (XLS 42 kb) Additional file 5: Movie S3. Imaging of the pulmonary valve in a representative wild type heart at E19.5 by OCT. A three-dimensional reconstruction of a representative wild type E19.5 heart is shown with the pulmonary valve highlighted in purple, followed by optical sections through the heart. The pulmonary valve is indicated in purple, and a three-dimensional reconstruction of the valve is shown. A still image of this 3D reconstruction is shown in Fig. 5a. (MOV 3438 kb) Additional file 6: Movie S4. Imaging of the pulmonary valve in a representative *Mbnl1* ^*∆E3/∆E3*^ heart at E19.5 by OCT. A three-dimensional reconstruction of a representative *Mbnl1* ^*∆E3/∆E3*^ E19.5 heart is shown with the pulmonary valve highlighted in purple, followed by optical sections through the heart. The pulmonary valve is indicated in purple, and a three-dimensional reconstruction of the valve is shown. A still image of this 3D reconstruction is shown in Fig. 5b. (MOV 3542 kb) Additional file 7: Figure S2. Color and pulsed Doppler ultrasound revealed valve regurgitation and atrial communication in *Mbnl1* ^*E∆3/∆E3*^ mice. (A) Sequential color Doppler ultrasound images show regurgitation across the aortic valve of an *Mbnl1* ^*∆E3/∆E3*^ mouse. Arrows indicate the direction of blood flow. LV = left ventricle, Ao = aorta, AV = aortic valve. Pulsed Doppler ultrasound revealed regurgitation across *Mbnl1* ^*∆E3/∆E3*^ (B) pulmonary, (C) aortic, and (D) mitral valves. Arrows indicate regurgitation peaks. Atrial communication was observed in some *Mbnl1* ^*∆E3/∆E3*^ mice by (E) color and (F) pulsed wave Doppler ultrasound. RA = right atrium, LA = left atrium, RV = right ventricle, LV = left ventricle. (PDF 8654 kb) Additional file 8: Figure S3. Scoring system for heart valve pigmentation. To evaluate the extent of pigmentation of the valves, a scoring system was developed in which each valve was given a score of 0 (no melanin), 1 (sparse melanin), 2 (moderate melanin), or 3 (abundant melanin). Representative inflow and outflow valves for each category are shown. (PDF 3893 kb)

## Abbreviations

AVC: Atrioventricular canal
CSPG: Chondroitin sulfate proteoglycan
DCT: Dopachrome tautomerase
DMP: Dorsal mesenchymal protrusion
E: Embryonic day
ECM: Extracellular matrix
ELISA: Enzyme-linked immunosorbent assay
EMT: Epithelial-mesenchymal transition
GAP: GTPase activating protein
MBNL: Muscleblind-like
OCT: Optical coherence tomography
OFT: Outflow tract
SPP1: Secreted phosphoprotein 1
TGFβ: Transforming growth factor β
TYRP1: Tyrosinase related protein 1
