# Supplementary figures and images for: Muscleblind-like 1 is required for normal heart valve development in vivo
Source: BMC Dev Biol. 2015 Oct 15;15:36. doi: 10.1186/s12861-015-0087-4 (PMC4608261; doi:10.1186/s12861-015-0087-4)

Figure S1

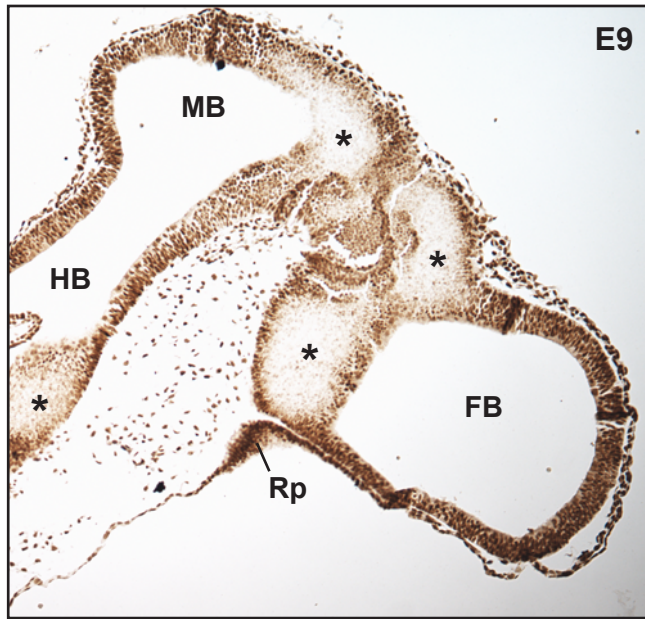

Supplement: Additional file 1: Figure S1. — MBNL1 expression in the embryonic brain. Immunohistochemistry with an anti-MBNL1 antibody was performed on sagittal sections from E9 wild type mouse embryos. Close-up of the head shows strong MBNL1 expression in the ectoderm lining the entrance to Rathke’s pouch (Rp), forebrain (FB), midbrain (MB), and hindbrain (HB) with little to no detectable staining within the interior encephalic tissues (highlighted by asterisks). A representative section from one of four embryos is shown. (PDF 1247 kb) [file 12861_2015_87_MOESM1_ESM.pdf]

Figure S2

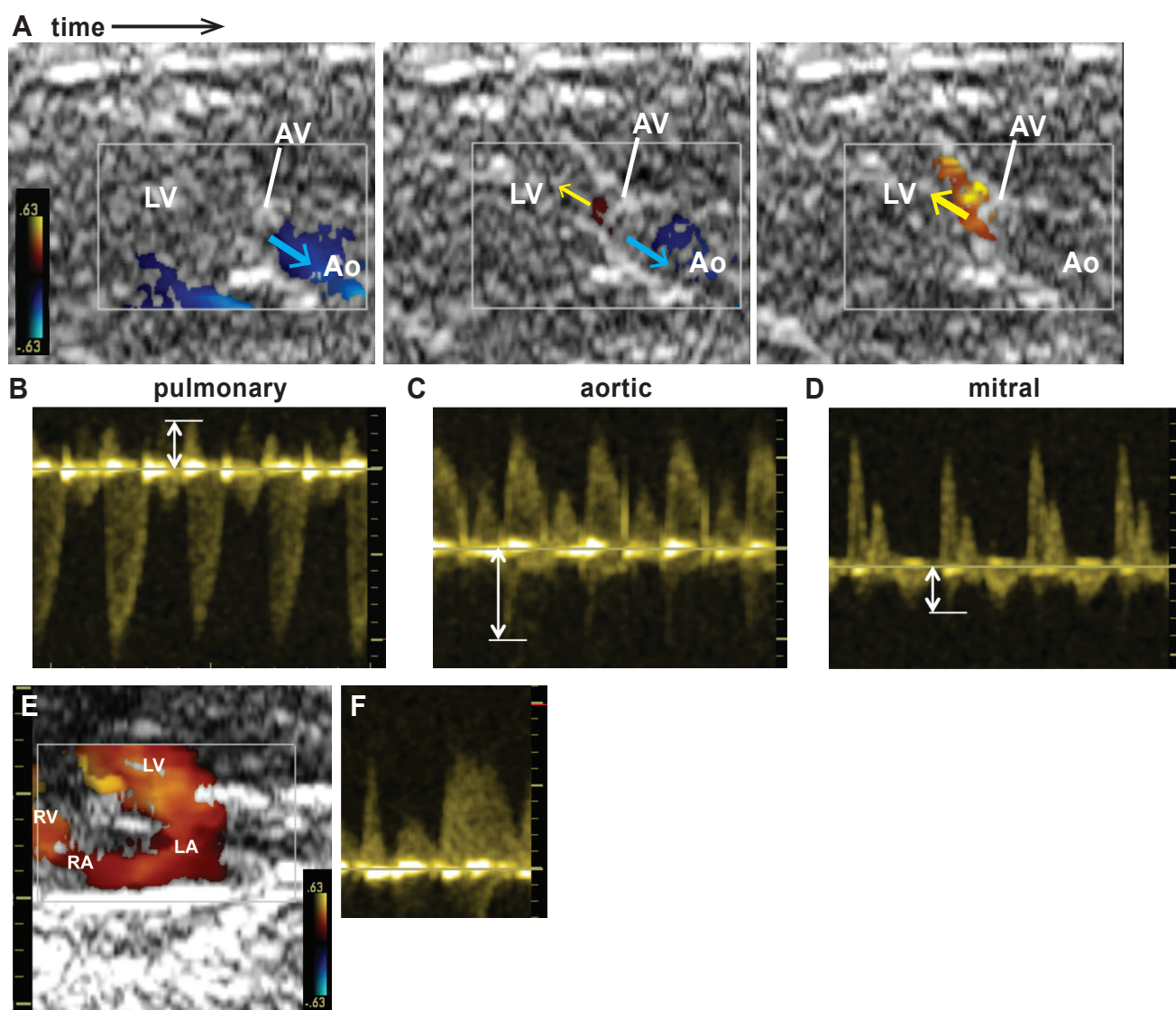

Supplement: Additional file 7: Figure S2. — Color and pulsed Doppler ultrasound revealed valve regurgitation and atrial communication in Mbnl1 E∆3/∆E3 mice. (A) Sequential color Doppler ultrasound images show regurgitation across the aortic valve of an Mbnl1 ∆E3/∆E3 mouse. Arrows indicate the direction of blood flow. LV = left ventricle, Ao = aorta, AV = aortic valve. Pulsed Doppler ultrasound revealed regurgitation across Mbnl1 ∆E3/∆E3 (B) pulmonary, (C) aortic, and (D) mitral valves. Arrows indicate regurgitation peaks. Atrial communication was observed in some Mbnl1 ∆E3/∆E3 mice by (E) color and (F) pulsed wave Doppler ultrasound. RA = right atrium, LA = left atrium, RV = right ventricle, LV = left ventricle. (PDF 8654 kb) [file 12861_2015_87_MOESM7_ESM.pdf]

Figure S3

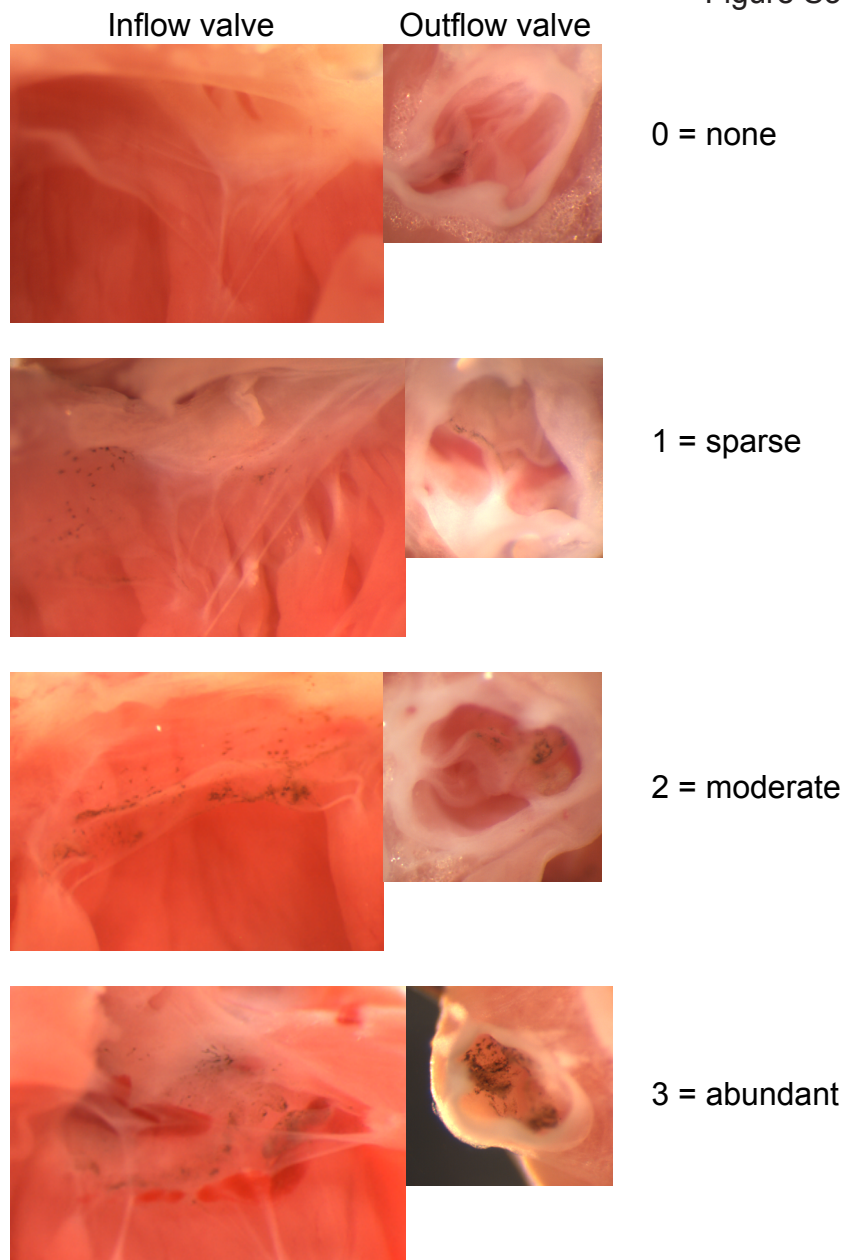

Supplement: Additional file 8: Figure S3. — Scoring system for heart valve pigmentation. To evaluate the extent of pigmentation of the valves, a scoring system was developed in which each valve was given a score of 0 (no melanin), 1 (sparse melanin), 2 (moderate melanin), or 3 (abundant melanin). Representative inflow and outflow valves for each category are shown. (PDF 3893 kb) [file 12861_2015_87_MOESM8_ESM.pdf]
